# Anatomical connectivity and the resting state activity of large cortical networks

**DOI:** 10.1016/j.neuroimage.2012.10.016

**Published:** 2013-01-15

**Authors:** D.A. Pinotsis, E. Hansen, K.J. Friston, V.K. Jirsa

**Affiliations:** a The Wellcome Trust Centre for Neuroimaging, University College London, WC1N 3BG, UK; b Aix-Marseille Université, Institut de Neurosciences des Systèmes UMR INSERM 1106, Faculté de Médecine, 27 Boulevard Jean Moulin, 13005 Marseille, France

**Keywords:** Large scale networks, Functional connectivity, Rest-state dynamics, Optimal community structure, Anatomical connectivity, Neural field equation

## Abstract

This paper uses mathematical modelling and simulations to explore the dynamics that emerge in large scale cortical networks, with a particular focus on the topological properties of the structural connectivity and its relationship to functional connectivity. We exploit realistic anatomical connectivity matrices (from diffusion spectrum imaging) and investigate their capacity to generate various types of resting state activity. In particular, we study emergent patterns of activity for realistic connectivity configurations together with approximations formulated in terms of neural mass or field models. We find that homogenous connectivity matrices, of the sort of assumed in certain neural field models give rise to damped spatially periodic modes, while more localised modes reflect heterogeneous coupling topologies. When simulating resting state fluctuations under realistic connectivity, we find no evidence for a spectrum of spatially periodic patterns, even when grouping together cortical nodes into communities, using graph theory. We conclude that neural field models with translationally invariant connectivity may be best applied at the mesoscopic scale and that more general models of cortical networks that embed local neural fields, may provide appropriate models of macroscopic cortical dynamics over the whole brain.

## Highlights

► Homogeneous connectivity matrices give rise to harmonic patterns. ► Localised modes reflect inhomogeneous coupling. ► Demonstration of a relationship between graph theory, anatomy and dynamics measures. ► Neural masses may be more appropriate than fields for large cortical network dynamics.

## Introduction

This paper is about modelling the dynamics in large scale brain networks with both realistic and analytic connectivity matrices. It focuses on the large scale structure of cortical dynamics and considers the effect of heterogeneities in connectivity in terms of the relationship between structural and functional connectivity (Honey et al., 2007; Zhou et al., 2006). The study of anatomical connectivity in the human brain – in parallel with the spatiotemporal activity of resting state networks – has attracted significant attention during recent years, e.g. (Arieli et al., 1996; Biswal et al., 1995; Damoiseaux et al., 2006; Smith et al., 2009; Vincent et al., 2007). Despite the inherent structure of this activity being well-known (engaging the posterior cingulate, precuneus, lateral parietal and elements of the prefrontal cortex), an understanding of how anatomical connectivity produces brain dynamics at various temporal and spatial scales is only partial. This is, in part, due to the lack of detailed biophysical data as well as computational power, both of which have only recently become available. In the past ten years, a wealth of data from tracing studies has revealed the complexity of anatomical connections in the macaque brain, while an aim over the next few years is to provide a full description of the connectivity of the human brain — the “connectome” (Biswal et al., 2010; Sporns et al., 2005).

Here, we use anatomical connectivity matrices and focus on the implications that their form might have for the dynamical repertoire of resting state activity and how this activity could be modelled. We assume that anatomical connectivity will predict certain aspects of functional connectivity (defined as the correlations among activity in different parts of the brain), and the dynamics of resting state activity; in other words, we assume that the dynamics of the resting state reflects some inherent aspect of anatomical connectivity. Our agenda was to establish the sorts of dynamics that could be seen under particular forms of anatomical connectivity and to relate them formally from basic principles.

Before detailed connectome data were available, simplifying assumptions about the connectivity needed to be made to study the network dynamics on the full brain scale. These assumptions included the translational invariance of the connectivity commonly referred to as homogeneous (e.g. Jirsa and Kelso, 2000). In particular for the temporal domain these assumptions proved to be powerful (Jirsa and Haken, 1996, 1997; Robinson et al., 1997), but naturally suffered when applied to the spatiotemporal domain. The symmetry present in the connectivity expressed itself as a constraint imposed upon the emergent brain dynamics. This led researchers to the proposal to use tractographic data as a connectivity skeleton of network models for the exploration of neural dynamics on the full brain scale (Jirsa et al., 2002). Here we were interested in exploring these connectivity constraints systematically. To address this, we use simulations to verify theoretically motivated characterisations of resting state dynamics and then use these characterisations to see whether homogeneity assumptions were justified when using realistic (DSI) connectivity matrices.

Our theoretical analyses focus on fixed-point attractor networks, where self-sustained oscillations are precluded (Jirsa and Ding, 2004) and, by definition, the network dynamics depend only upon the anatomical or structural connectivity matrix. This matrix allows us to express the dependence of spatiotemporal dynamics, arising from the collective activity over the network nodes, in terms of a few sufficient modes, which correspond to the dominant patterns of cortical activity observed at rest. To allow for some mathematical treatment, we simplify the mean field formulation of Brunel and Wang populations to a stable fixed-point attractor absorbing both effects of excitatory and inhibitory activity. Attractor networks have been demonstrated to show non-oscillatory instabilities for excitatory coupling with random connection topology and constant identical time delay for all couplings (Feng et al., 2006; Jirsa and Ding, 2004). There are currently no results available extending this work to multiple time delays. For our purposes, we focus on the (sub)set of non-oscillatory solutions arising at instabilities. This assumption does not exclude complex dynamics within a population as demonstrated by Brunel and Wang (2001) who considered mean fields of populations of spiking neurons based on the integrate-and-fire neuronal model. It is the mean field of the population activity that demonstrates the attractor state, which is the level of description chosen here. This result generalizes to more complex situations under certain conditions on the dispersion of neural activity and noise (Assisi et al., 2005; Jirsa, 2007; Stefanescu and Jirsa, 2008). In short, our focus is on fixed-point solutions of mean field formulations of Brunel and Wang neuronal populations. Noise induced in a population of spiking neurons will change the spiking dynamics, but the mean field will have a particular constant value as long as all other characteristics stay the same and this noise is small. Jirsa and Ding showed that inhibition is crucial in order to generate a delay-induced oscillatory instability for the network. However, this result holds only for random connectivity and when delay is the same across the whole network.

Our paper follows a series of studies focusing on the relationship between the structural properties of the brain and the nature of dynamics on brain networks. In particular, we take up the themes motivated by a graph theoretical approach to complex systems (Newman, 2003; Watts and Strogatz, 1998). This approach started with the paper of Watts and Strogatz — who studied the anatomical connectivity of *Caenorhabditis elegans*. Graph theory has been used to delineate significant traits of neuroanatomical networks (He and Evans, 2010; Stam and Reijneveld, 2007), such as their small-world architecture and derive measures that can be associated with dysfunction (Ponten et al., 2007; Schindler et al., 2008). Furthermore, graph theory has been used to identify plausible brain systems in a mind-wandering state, often called a default mode network, e.g. (Buckner et al., 2008; Raichle et al., 2001). Recent research has also focused on describing pathological brain states by associated graph theoretical measures, reflecting clinico-pathological processes (Bassett and Bullmore, 2009; Guye et al., 2010) and has used neural fields to analyse large scale cortical networks (Gray and Robinson, 2007, 2009; Gray et al., 2009; Henderson and Robinson, 2011; Robinson et al., 2008, 2009) and neural masses to model local sources (Jansen and Rit, 1995; Lopes da Silva et al., 1974; Van Rotterdam et al., 1982) or construct neurocognitive networks (Dhamala et al., 2007). We here focus on notions such as community structure, to motivate similar approximations of translational invariance (Amari, 1977; Breakspear et al., 2006; Coombes, 2010; Deco et al., 2008; Robinson et al., 2003; Wilson and Cowan, 1972) and consider the role of inhomogeneities in producing endogenous brain dynamics. As pointed out by a reviewer, there is a huge literature on the analysis and interpretation of fields in the physics literature — ranging from optics and electromagnetics to acoustics and quantum fields. Also, it is well known that localised modes will be produced by inhomogeneities (e.g., impurities in condensed matter), and that translation-invariant systems will have plane eigenmodes (this goes back to Nother in the early 1900s, see e.g. Bloch, 1929). Our focus is on heterogeneities of the sort assumed in neural field theory and whether they have a similar effect as “matrix impurities”. For instance, in condensed matter physics, impurities act only locally which is different from the combination of long-range interactions and local homogeneous kernels we consider here.

This paper comprises three sections: in the first, we discuss the anatomical data that form the basis of our investigation and introduce some notions from graph theory that we will use to characterise inhomogeneities in realistic and analytic connectivity matrices. In the second, we formulate cortical dynamics in terms of an integro-differential equation, thereby providing an analytical characterisation of resting state activity; that is dominated by eigenmodes of the connectivity matrix. We also consider a homogeneity approximation to the connectivity matrix; namely, we replace a sparse connectivity matrix by translationally invariant coupling (modelling lateral interactions on the cortical sheet), and provide a mathematical explanation for the emergence of damped spatially periodic patterns. In the third section, we turn to numerical investigations and study the emergent dynamics from various connectivity configurations and exemplar matrices. We demonstrate the relationship between graph theory measures of clustering and principal eigenmodes. In addition to our focus on symmetric (undirected) connectivity matrices, we also discuss the importance of asymmetries and coherent fluctuations, such as those observed in resting state fMRI by exploiting connectivity configurations including short range excitatory and inhibitory effects. Finally, we used the theoretical predictions to assess the adequacy of translationally invariant connectivity matrices to explain the principal patterns of activity associated with realistic anatomical connections.

## Large scale brain networks

The analysis of large connectivity datasets, using graph theory, considers brain networks as weighted graphs, whose nodes represent cortical sources of measurable activity and whose edges correspond to anatomical connections. Each of these edges can be associated with a number corresponding to the weight that characterises the strength of the relevant connection; for example, as obtained through tracing studies. These weights then form a matrix, which describes anatomical connectivity.

### Diffusion spectrum imaging matrix

**Fig. 1:**
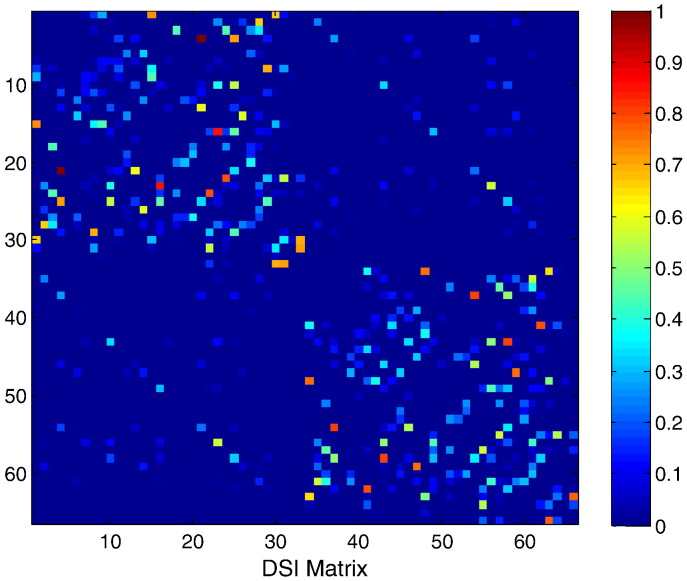
Neuroanatomical connectivity matrix obtained by human DSI data. Different colours depict connectivity strengths between 0 and 1.

We use an anatomical connectivity matrix denoted by *κ* obtained via diffusion spectrum imaging (DSI) and white matter tractography (Hagmann et al., 2008). This matrix consists of *N =* 66 nodes representing both hemispheres of the human brain (see Fig. 1).

The matrix entries *κ*_*ij*_ are the corresponding connection weights that range from 0 to 1 (in an arbitrary scale) and are based on tract density. The upper left and lower right quadrants represent intrahemispheric connections. Elements of a band structure in this matrix reflect prominent fibre-tracts connecting homologous regions in the two hemispheres. Since the DSI technique yields non-directional connections *κ*_*ij*_ = *κ*_*ji*_. With each entry *κ*_*ij*_ ∈ *κ* we can associate two regions *x*_*i*_ and *x*_*j*_ corresponding to source and target regions respectively. We will denote the space spanned by all such regions by *W*, namely *W* = {∪ *x*_*l*_}_*l* = 1_^*N*^. The matrix *κ* can be represented as a function in a two-dimensional space *κ*(*x*,*y*), where the variables *x* and *y* parameterise the location of target and source regions respectively, assuming, for simplicity, one-dimensional cortical manifolds.

### Optimal community structure, modularity and clustering coefficient

Here, we briefly review certain notions from graph theory, used in this paper, that have proven very useful for a quantifying brain network topology. For the evaluation of graph theoretic quantities we used the Brain Connectivity Toolbox (Rubinov and Sporns, 2010), (see also Ghosh et al., 2008; Honey et al., 2007). First, we consider the *clustering coefficient*: this was introduced in Watts and Strogatz (1998) and is defined as the ratio of all extant connections between a node's neighbours divided by all possible such connections. It is based on a partitioning of a graph into triangles that serve as basic motifs of network structure and can be expressed by the formula $\bar{\gamma_{a}}=\varepsilon_{a}\gamma_{a}$. In the latter formula, $\gamma_{a}=2p_{a}^{-1}{\left(p_{a}-1\right)}^{-1}\delta_{a}$ where *p*_*a*_ and *δ*_*a*_ are the degree of and the number of triangles attached to a node *a* respectively and *ε*_*a*_ is the average intensity of triangles at this node. For more details, we refer the reader to Onnela et al. (2005). The (local) clustering coefficient indicates the embeddedness of a single node, in terms of the nodes to which it is connected. Later, we examine the ability of the (local) clustering coefficient to predict patterns of resting state dynamics; in particular the eigenmodes of cortical activity.

**Fig. 2:**
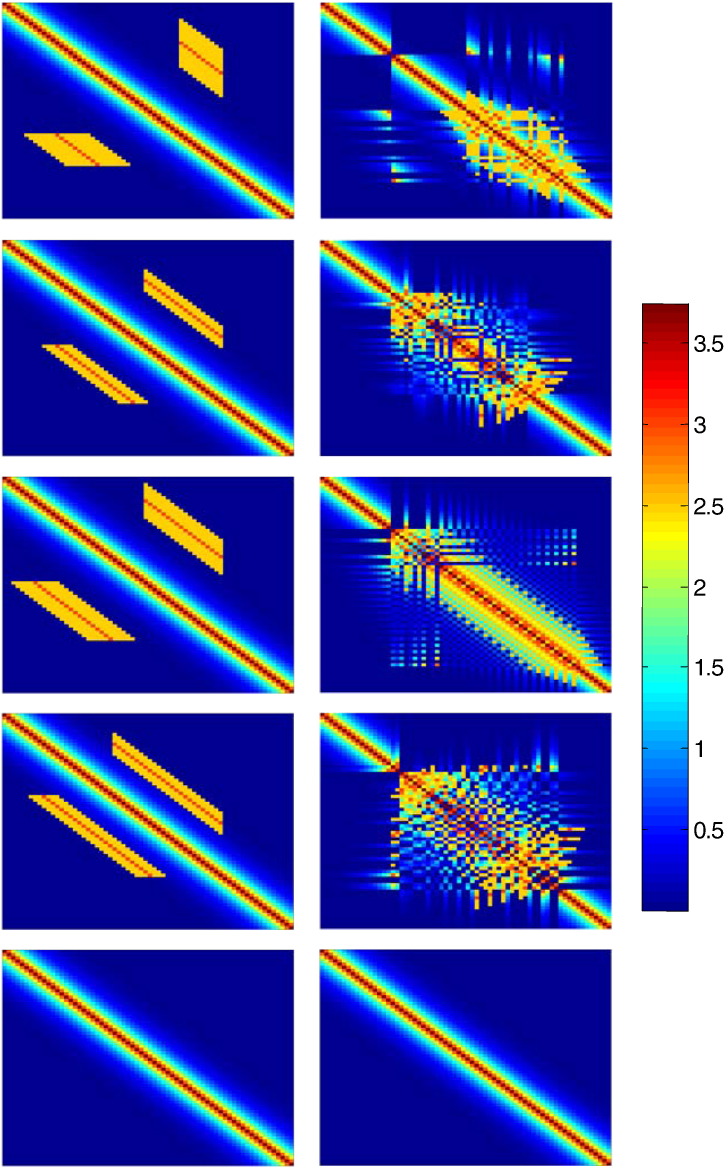
Exemplar connectivity matrices (left column) and their reordered versions in terms of optimal communities (right column). Matrices in the left column are diagonal-constant and symmetric and can be considered as perturbations to the matrix depicted in the bottom of the left column(exponential synaptic footprint) which is a standard matrix used in neural field theory; these matrices include both a translationally invariant part (around the main diagonal) and inhomogeneous two-point connections. Jirsa (2009) has shown that such matrices can provide a general formulation of neural field dynamics including both local homogeneous and long-range heterogeneous coupling. The matrices of the right column motivate a reordering of the realistic DSI matrix considered later that allows assessing homogeneity assumptions for whole-brain dynamics (see last section). In the bottom panel, we see an example of a translationally invariant connectivity matrix *κ*(*x*,*y*), with $\kappa \left(x,y\right)=\frac{s^{-\left|x-y\right|/\sigma }}{2\sigma }$. This kernel is called an exponential synaptic footprint and has often been used in the literature to account for excitatory and inhibitory interactions.

Another widely-used graph theoretical metric is *modularity*, which characterises the organisation of a complex network into sub-modules or communities; these are distinguished by a high degree of connections between nodes of the same module, in comparison with the degree of connections of these nodes to network elements outside this module, reflecting a natural segregation within the network (Newman, 2003). Modular organisation has been assumed to indicate groups of anatomically or functionally associated nodes that work together to achieve behavioural functions with some degree of independence. Modularity (functional segregation) endows the network with flexibility so that it can adapt one community (functionally segregated neuronal system) without affecting others that subserve other specific functions, see e.g. (Boccaletti et al., 2006). In mathematical terms, modularity is evaluated in terms of a cost function (Newman and Girvan, 2004) expressing the number of edges falling within a community minus their expected number in a random network

$$C=\sum_{a}\left(\zeta_{\mathrm{a\beta}}-{\left(\sum_{\beta }\zeta_{\mathrm{a\beta}}\right)}^{2}\right)\text{,}$$

where *ζ*_*aβ*_ is the fraction of edges in the network that link communities *a* and *β*. Newman and colleagues realised that values of *C* > 0.3 indicate a significant community structure and proposed an efficient algorithm for optimising *C* over all possible subdivisions of a network. An *optimal community structure* of the network is obtained by its division into several communities, so that the number of edges within the same community is maximised over all communities, while the number of between-community edges is minimised. In this paper, we use the reordered connectivity matrices depicted in the right column of Fig. 2, that juxtapose nodes from the same community, to establish the existence of local (homogenous) cliques that may conform to homogeneity assumptions (see below). We consider symmetric Toeplitz matrices involving inhomogeneous connections and their reordered versions according to optimal communities. We note that the latter versions favour the appearance of block structures around the main diagonal and motivate a reordering of the realistic DSI matrix to which we will come back later (please see top right panel of Fig. 8). In the last section, we will also use these matrices to exemplify the relation between resting state activity, anatomical modes and graph theoretical measures.

### Summary

In summary, this section has introduced the basic notions of connectivity matrices or kernels that define a graph theoretic representation of anatomical coupling between cortical regions in the brain. We have introduced the notions of clustering and optimal community structure. These reflect the tendency of nodes to cluster together and the modularity of that clustering respectively. In what follows, we will examine the sorts of dynamics that might be expected for any given anatomical connectivity or graph.

## Cortical dynamics and anatomical connectivity kernels

In this section, we introduce the mathematical formalism for describing neural dynamics on anatomical connectivity structures. We employ an integro-differential form for cortical activity following neural field theory (Gray and Robinson, 2007, 2009; Jirsa and Haken, 1996, 1997; Robinson et al., 2008, 2009). We then briefly motivate equilibrium solutions of the ensuing dynamics, in which we can ignore conduction delays. This allows us to use the standard linear stability theory to link fluctuations in resting state activity to the underlying anatomical connectivity, in terms of patterns or eigenmodes of the anatomical connectivity. Finally, we examine the special case of neural field models in which anatomical connectivity is homogeneous and examine some of the spatial characteristics of resting state fluctuations that would be anticipated under this approximation.

### An integro-differential equation for cortical dynamics

Following the theory of neural fields with constant coefficients in continuous media, we consider the connectivity kernel *κ*(*x*,*y*) as a scalar function in a two-dimensional Euclidean space. This allows us to describe the propagation of activity in large cortical networks via an integro-differential equation. In this formulation, the connectivity kernel *κ*(*x*,*y*) is often of a particular homogeneous or translationally invariant (stationary) form. It should be emphasised that the differential formulation of neural fields with constant coefficients in continuous media only applies when *κ*(*x*,*y*) has a homogeneous form.^1^ Jirsa (2009) describes a general form of a neural field theoretical equation and splits it into two components, one with a translationally invariant kernel characteristic for local coupling and one with a translationally variant kernel for long range coupling. The general theory of neural fields involves an integro-differential equation prescribing the time evolution of cortical activity at a certain location and time point. It assumes that activity at time *t* at a location *x* is characterised by some quantity *q*(*x*,*t*) representing e.g. average depolarisation and simply says that this activity evolves as a result of postsynaptic filtering (with decay constant *τ*) of afferent input,

(1) $$\left(\partial_{t}+\tau \right)q\left(x,t\right)=H\left(f\left(q\right)\right)\left(x,t\right)\;x\in W$$

where *H* = *H*(*f*(*q*)) is incoming activity to point *x* of a region *W* at time *t* from all regions connected to it and is given by

(2) $$H\left(f\left(q\right)\right)\left(x,t\right):=\int_{W}\kappa \left(x,y\right)f\left(q\left(y,t\right)\right)dy\text{.}$$

Here *f*(*q*) denotes input obtained after passing the activity of an afferent (source) population *q*(*y*,*t*) through a sigmoid nonlinearity $f\left(q\right)=\frac{1}{1+\exp\left(a\left(q-h\right)\right)}$, and *a*, *h* are an effective gain and firing threshold respectively, which summarize ensemble features of the source population.

Eq. (1) is the deterministic version of the well-known Amari neural field equation (Amari, 1974). For our numerical analyses below, we used its stochastic version based on the following Langevin equation

$$dq\left(x,t\right)=-q\left(x,t\right)+H\left(f\left(q\right)\right)\left(x,t\right)+\varepsilon^{1/2}w\text{.}$$

The function *w* is a spatially uncorrelated Wiener process:

$$\begin{array}{l} \langle w\left(x,t\right)\rangle =0\\ \langle w\left(x,t_{a}\right)w\left(y,t_{b}\right)\rangle =\delta \left(x-y\right)\delta \left(t_{a}-t_{b}\right) \end{array}$$

where *ε* is a small number so that noise is low.

It should be noted that Eq. (1) lacks any explicit propagation delays; and is formally equivalent to a system of coupled oscillators. The advantage of employing an integral formalism will become apparent in the next section, when we will discuss the relation between resting state activity and dominant patterns of structural connectivity. However, time delays associated with signal transmission cannot, in principle, be omitted in any serious stability analysis of the brain network's dynamics. There are though some special cases, in which delays may be ignored: first, the existence of the equilibrium solutions that are independent of time delays. The linear stability of networks with equilibrium solutions is generally not independent of time delays, unless we consider only networks with fixed-point attractors, see for instance (Deco et al., 2009). (Jirsa and Ding, 2004) have shown that fixed-point networks with a common time delay and random connectivity display two types of bifurcations, an oscillatory and non-oscillatory instability. The latter occurs for purely excitatory connections and is not restricted to random matrices but applicable to all connectivity configurations. We here focus on dynamics arising from matrices including purely excitatory connections where the non-oscillatory instability is independent of the time delay (under the assumption of a single delay discussed above).

### The repertoire of resting state activity and eigenmodes

In this section, we show that the repertoire of resting state activity is spanned by the eigenmodes of the anatomical connectivity matrix. Assuming that fluctuations around the resting state result in transient non-Turing patterns, namely patterns that decay back to the resting state with an associated Lyapunov exponent *λ* — and substituting $q\left(x,t\right)=u_{0}\left(x\right)+\sum_{l}e^{\lambda_{l}t}v_{l}\left(x\right)$ into Eq. (2), we obtain an eigenvalue equation

(3) $$Fv_{l}\left(x\right)=\lambda_{l}v_{l}\left(x\right)$$

where the operator *F* is given by *F* = *H* − *τ* and the firing rate function has been approximated with a linear function of the gain of the sigmoid function above: in all subsequent discussion, without loss of generality, we assume *f*(*u*_0_) = 0. This implies that cortical activity at rest is given by a superposition of eigenmodes of the operator *H*. Assuming a decomposition of the connectivity matrix *κ*(*x*,*y*) in terms of its eigenmodes denoted by

(4) $$\kappa \left(x,y\right)=\sum_{l=0}^{N}L_{l}V_{l}\left(x\right)V_{l}\left(y\right)$$

and that (without loss of generality) *λ*_*l*_ *= L*_*l*_, we find that *v*_*l*_(*x*) = *V*_*l*_(*x*) for all *l*. Therefore, the (eigen) spectrum of the anatomical connectivity matrix *κ*(*x*,*y*) identifies the dynamics of cortical activity at rest (see also Deco et al., 2009, 2010; Ghosh et al., 2008). The set of eigenmodes with the smallest real eigenvalues correspond to those patterns in the space *W* that will be seen during transient fluctuations around the resting state. In other words, these are the patterns that decay most slowly following a perturbation; the remaining patterns (eigenmodes) dissipate almost immediately (Robinson et al., 1997, 2001). The rate at which the system returns to its resting state after a perturbation is characterised by the corresponding eigenvalue with an associated time constant:

(5) $$\tau_{l}=-1/\lambda_{l}$$

where the *ι*-th eigenvalue *λ*_*l*_ is given by

(6) $$\lambda_{l}=\int_{W}V_{l}\left(x\right)FV_{l}\left(x\right)dx\text{.}$$

The key thing to notice here is that, in the resting state, most structural modes will decay rapidly to zero and their contribution to resting state dynamics will be negligible. In particular, the resting state solution will be dominated by the first few eigenvalues and the corresponding modes will reveal those anatomical patterns that predominate in patterns of resting state fluctuations. This provides a rationale for reducing the dimension of the solution at resting state by ignoring higher order modes: put simply, the results above mean that we can express neuronal activity as a mixture of patterns or modes, where these modes can be derived from any given anatomical connectivity matrix or kernel (as its eigenvectors). Furthermore, we only need to consider a small number of patterns that are associated with small (negative) eigenvalues, because these unstable modes will dominate the dynamics.

### An approximation of translational invariance

Realistic connectivity DSI matrices such as the matrix of Fig. 1, are usually quite sparse and reflect a high degree of inhomogeneity in cortical connections.^2^ However, translationally invariant matrices with a characteristic spatial decay of lateral synaptic densities have often served as a first approximation to *κ*(*x*,*y*) (e.g. Breakspear et al., 2006; Jirsa and Haken, 1997; Nunez, 1995; Robinson et al., 1997), see the bottom panel of Fig. 2 for an example of such a matrix.

As mentioned above, an *approximation* of a large scale connectivity matrix with a translationally invariant matrix has often been used to describe cortical activity. This amounts to replacing the realistic connectivity kernel in Eq. (1) by a parsimonious analytic approximation of the sort used in the differential formulation of neural field models in continuous media. Dynamics resulting from such an approximation will, of course, differ from realistic resting state dynamics: even if we choose the nonlinearity in Eq. (2) carefully, so that both the realistic as well as the homogenous network have the same fixed-point solution; neglecting inhomogeneities in the connectivity results in omitting important features of cortical activity.

However, it may be the case, at least locally, that the real connectivity can be considered translationally invariant. Furthermore, community structure could provide a way of identifying local homogeneity in realistic connectivity matrices. In particular, a reordering of the connectivity matrix to reveal community structure, generally produces block-structures and discloses local cliques (see Fig. 2). The existence of these communities is potentially important because they can be used to motivate the assumption of a translationally invariant connectivity matrix: namely, that the regions across which activity propagates form a one-dimensional manifold on which local synaptic connection density decays in a homogenous fashion (e.g. exponentially). In other words, the underlying assumption is that each node in the network is connected to its neighbours by means of an exponentially decaying synaptic density. Cortical activity will then propagate across the cortical network, induced by successive excitations of neighbouring regions as a result of local diffusion.

In what follows, we adopt an assumption such as the one described above, to consider the nature of spatiotemporal dynamics that would arise within a single community or clique.

### Translationally invariant connectivity yields spatially periodic activity

In what follows, we will limit ourselves to homogeneous or translationally invariant connectivity matrices that can give rise to spatially periodic activity; such an approximation *restricts* the dynamical repertoire of resting state activity to decaying spatially periodic patterns, something that could preclude localised dynamics^3^; a theme to which we return in the next section. Conversely, spatially periodic patterns do not necessarily imply a periodic connectivity matrix and in fact appear as a result of local diffusion. We show that the eigenmodes of a large class of translationally invariant matrices are damped patterns characterised by spatial periodicity (see also Robinson, 2006) and later confirm our results through numerical simulations.

Assuming that *κ*(*x*,*y*) = *κ*(|*x* − *y*|) is a function of the spatial separation of cortical sources and considering perturbations around a fixed-point, we take the Fourier-Laplace transform of Eq. (1) (see e.g. Grindrod and Pinotsis, 2011; Jirsa and Haken, 1996; Pinotsis and Friston, 2011),

(7) $$\left(\lambda +\tau \right)Q\left(k,\lambda \right)=K\left(k\right)F\left(k,\lambda \right),\;\lambda \in C,k\in R$$

where, for notational clarity, we will use lower case letters for time-varying quantities and upper case latter for their transform, namely *Q*(*k*,*λ*) is the Fourier–Laplace transform of the depolarisation

(8) $$\begin{array}{c} Q\left(k,\lambda \right)=FLT\left(q\left(x,t\right)\right)\\ =\int_{-\infty }^{\infty }dx\int_{0}^{\infty }dtq\left(x,t\right)e^{-ikx+\lambda t}\text{.} \end{array}$$

*K*(*k*),*F*(*k*,*λ*) are the Fourier and Fourier–Laplace transform of the connectivity kernel and depolarization-firing function *κ*(*x*,*y*) and *f*(*q*)(*x*,*t*) respectively. As a general illustrative example of translationally invariant connectivity matrices, we now consider matrices with a Fourier transform of a *rational* form^4^ (see also Robinson, 2006)

(9) $$K\left(k\right)=\frac{Ak^{n}+Bk^{n-1}+\cdot \cdot \cdot +\Gamma }{ak^{m}+\beta {k^{m}}^{-1}+\cdot \cdot \cdot +\gamma }\;m>n\text{.}$$

A widely used connectivity kernel with a rational Fourier transform, the so-called exponential synaptic footprint (Jirsa and Haken, 1996) $\kappa \left(\left|x-y\right|\right)=\frac{e^{-\left|x-y\right|/\sigma }}{2\sigma },\sigma >0\text{,}$ is depicted in Fig. 2 (bottom), with Fourier transform

(10) $$K\left(k\right)=\frac{1}{1+k^{2}\sigma^{2}}\text{.}$$

Using Eqs. (9) and (7), the *nonlocal* integro-differential Eq. (1) reduces to a *local* partial differential equation (PDE) of the following general form

(11) $$\begin{array}{l} \left(a'{\partial_{x}}^{m}\partial_{t}+\beta '{\partial_{x}}^{m-1}\partial_{t}+\ldots +\tau \left(\gamma '{\partial_{x}}^{m}+\delta '{\partial_{x}}^{m-1}+\ldots \right)+\varepsilon '\right)q=\\ \left(A'{\partial_{x}}^{n}+B'{\partial_{x}}^{n-1}+\ldots +\Gamma '\right)f\left(q\right) \end{array}$$

where ∂ _*x*_ and ∂ _*t*_ denote partial derivative operators with respect to *x* and *t* respectively. The constants *a*',*β*',… and *A*',*B*',… are uniquely defined in terms of the constants *a*,*β*,… and *A*,*B*,… after multiplying both sides of Eq. (7) by the denominator of the right hand side of Eq. (9) and taking the inverse Fourier–Laplace transform and are constrained by the requirement that the resulting solution decays back to a steady-state. A particular instance of Eq. (11) can be obtained using expression (10). It turns out that λ∈R and there is no frequency dependence in the observed dynamics. The physical regime where this approximation is valid includes very low frequencies. This conforms to our prior assumption about focusing on the (sub) set of non-oscillatory solutions arising at instabilities. The exponential synaptic footprint has often been used in the literature to allow the application of standard techniques in PDE theory to be used in the analysis of neural fields; since, in the presence of delays, it gives rise to wave equations that describe electrical activity propagating on the cortex^5^ (e.g. Amari, 1974; Beurle, 1956; Breakspear et al., 2006; Coombes et al., 2007; Freeman, 1972; Jirsa and Haken, 1996; Nunez, 1995; O'Connor and Robinson, 2004; Robinson et al., 2003; Wilson and Cowan, 1972). In this case, Eq. (11) reduces to

(12) $$\left(\partial_{t}-e\partial_{\mathrm{xx}}\partial_{t}-g\partial_{\mathrm{xx}}+g/e\right)q=f\left(q\right)$$

where the constants *e* and *g* are defined in terms of *τ* and σ. Letting (∂ _*t*_ + *g*/*e*)*q* = *p*, Eq. (12) becomes (− *e* ∂ _*xx*_ + 1)*p* = *f*(*q*), which is the limit of a wave equation describing propagation of afferent input *p* when delays are neglected, see e.g. (Jirsa, 2009; Pinotsis et al., 2012). The function *f*(*q*) appearing in Eq. (11) or (12) drives this afferent input and *q* in this function denotes the depolarization of the source region or population. It is well known that solutions to linear partial differential equations with constant coefficients such as Eq. (11) or (12), can be expressed as superpositions of periodic modes $\sum_{l}c_{l}e^{ik_{l}x}$, whose wavenumbers *k*_*l*_ can be obtained from the corresponding dispersion relation (for a relevant discussion see Pinotsis and Friston, 2011). This relation prescribes the spectral responses of the brain networks and – for the case of an abstract network described by the general Eq. (11) – reads

(13) $$a\left(\lambda +\tau \right)k^{m}+\beta \left(\lambda +\tau \right)k^{m-1}+\cdot \cdot \cdot +\gamma \left(\lambda +\tau \right)-Ahk^{n}-Bhk^{n-1}-\cdot \cdot \cdot -\Gamma h=0$$

where *h* = *f*′(*u*_0_) is the gain associated with the depolarization-firing rate function. For the case of an exponentially decaying synaptic density, Eq. (13) yields

(14) $$\sigma^{2}\left(\lambda +\tau \right)k^{2}+\lambda +\tau -h=0$$

which has roots $k_{12}=\pm \sigma^{-1}\sqrt{h{\left(\lambda +\tau \right)}^{-1}-1}$. This gives rise to two modes, propagating in opposite directions with the same decay rate *λ*. Combining Eq. (13) with the fundamental theorem of algebra we conclude that – in the general case of a connectivity of the form of Eq. (9) – fluctuations around the resting state will be a superposition of *m* damped modes (Robinson et al., 1997, 2001), whose wave numbers are determined by the connectivity parameters prescribed by the relevant dispersion relation – namely Eq. (13).

### Summary

In summary, network activity at resting state resulting from a translationally invariant connectivity matrix of the general form of Eq. (9) (and under the assumption of fixed-point dynamics of mean field formulations), could exhibit spatial periodicity^6^ consisting of damped spatially periodic modes with wave numbers corresponding to roots of Eq. (13). In other words, to the extent that homogenous coupling can be assumed over the cortical manifold (or a local cortical clique), resting state fluctuations should conform to cortical activity with spatial periodicity and scale determined uniquely by the extent and density of lateral anatomical connections. Clearly, the extent to which this is true depends upon the degree to which lateral connectivity is actually homogenous and the size of the cortical manifold relative to this connectivity. In the final section, we look at the failures of the homogeneity assumption using numerical solutions to the equations above.

## A phenomenological characterisation of emergent dynamics

In this section, we first focus on patterns emerging from perturbations around the resting state and the eigenmodes associated with each connectivity kernel. We will relate these eigenmodes to the (graph theoretic) clustering coefficient over nodes. We consider connectivity configurations for general inhomogeneous or homogenous connectivity configurations and investigate the correspondence between the associated functional and structural connectivity. We conclude with an analysis of non-symmetric (directed) connectivity and, in particular, a simulation of resting state activity using realistic (DSI) connectivity matrices. Our objective here was to look for evidence of damped periodic modes in communities of nodes, identified using graph theory, of the sort that would motivate the application of homogenous approximations to the connectivity of large-scale cortical dynamics.

### The resting state, principal modes and clustering coefficient

**Fig. 3:**
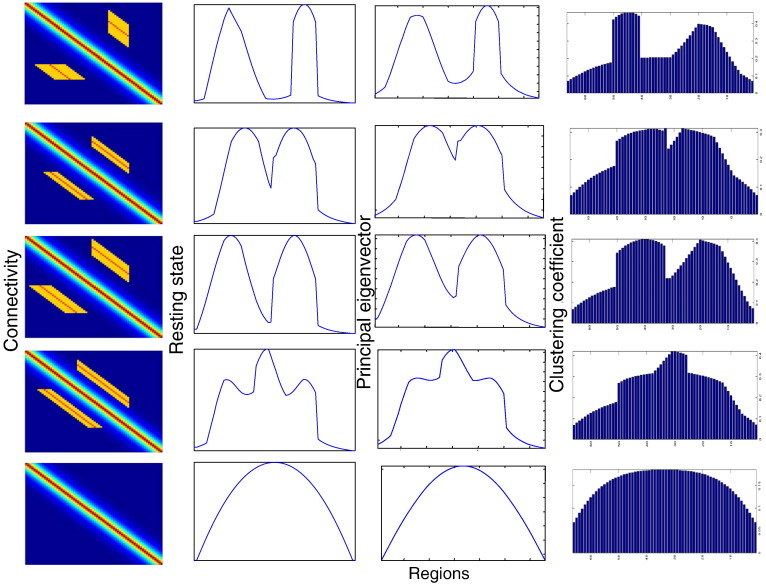
Exemplar anatomical connectivity matrices (first column), resting state (second column), principal eigenvector of anatomical connectivity (third column), and clustering coefficient of anatomical connectivity (fourth column).

By configuring the connectivity parameters so that each fixed-point network approaches the verge of instability, we integrate the neural field Eq. (2) with white noise input until an equilibrium state is reached to provide a plausible solution for resting state dynamics. For each simulation we assumed random initial and zero flux boundary conditions and scaled the connectivity matrix to ensure that at least one (real part) eigenvalue approached zero — this ensured realistic dynamics that did not dissipate quickly and can be considered a form of organised criticality. We solved Eq. (1) using an integrator scheme with time bins of 0.01 s, where the resting time series comprised of 2001 samples. The spatial discretization of the matrices with an analytical form is the same as for the DSI matrix. When taking eigenmodes of the exemplar connectivity matrices of Fig. 2, we find – as expected from the Centre Manifold Theorem outlined in the previous section – that the principal eigenvector coincides with the principal resting state pattern (modulo a phase difference of ± *π* depending on the sign of the principal eigenvector). The principal eigenmode of the (simulated) resting state activity was numerically evaluated by simply integrating the system to a steady state solution. In principle, this furnishes the same mode as the first eigenvector of the sample covariance or functional connectivity matrix (because this mode dominates the sample fluctuations). This covariance matrix corresponds to the functional connectivity matrix. This similarity confirms the theoretical predictions of the previous section — suggesting that there is a close correspondence between the eigenmodes of anatomical connectivity and the dominant modes of dynamics that result from fluctuations around an equilibrium solution. Below, we evaluate clustering coefficients and other graph theoretic measures for both simulated and realistic (DSI or CoCoMac) matrices. Similar to the case of DSI matrices where each entry contains a value corresponding to the strength of connections between two regions obtained through white matter tractography, the corresponding values for the simulated matrix of the bottom of Fig. 3 are given by the function $\kappa \left(x,y\right)=\frac{e^{-\left|x-y\right|/\sigma }}{2\sigma }$ where *x* and *y* are the coordinates corresponding to regions *x* and *y* (and the ordering of the regions follows the DSI matrix of Fig. 1).

Crucially, we observed that the eigenmodes of the anatomical connectivity matrix are very similar to the pattern of (local) clustering coefficients associated with the connectivity kernel (see Fig. 3). Recall that the local clustering coefficient reports the degree of embeddedness of any particular node in its cluster or community. This result should come as no surprise, if we consider that the integro-differential equation, we used to simulate activity, describes a series of identical microscopic oscillators connected to each other: for homogeneous or random initial conditions, we expect that resting activity will be dominated by oscillators that are densely interconnected and form local (highly embedded) cliques. In short, assuming that delays do not alter the stability properties of the system considered here, the underlying anatomical structure (expressed in terms of the clustering coefficient) determines the patterns of fluctuations in the resting state.

### Localised eigenmodes versus the neural field approximation

**Fig. 4:**
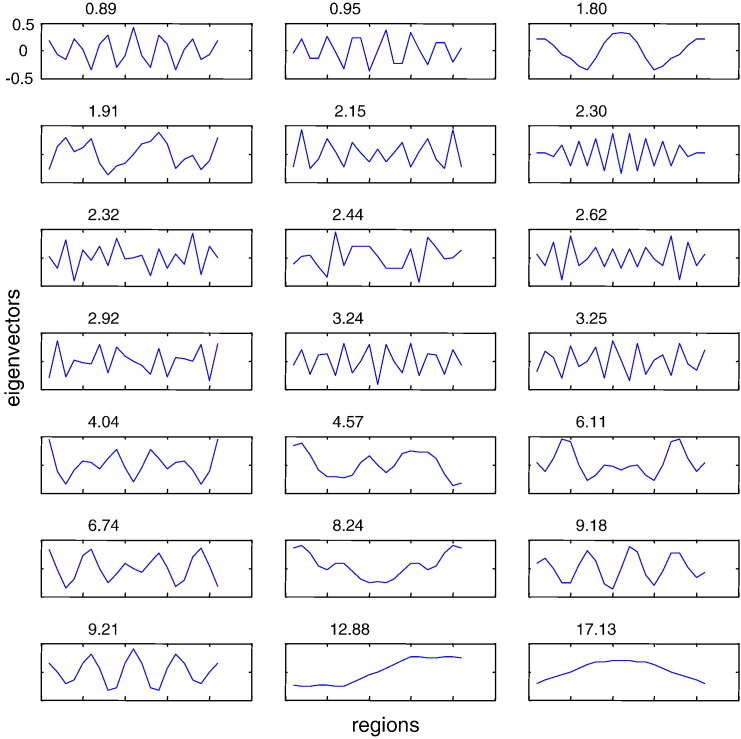
Full spectrum of periodic modes for a translationally invariant and symmetric connectivity matrix and corresponding eigenvalues (above each plot). Notice the dominant mode at the right bottom corner resembling the mode at the bottom of Fig. 3.

**Fig. 5:**
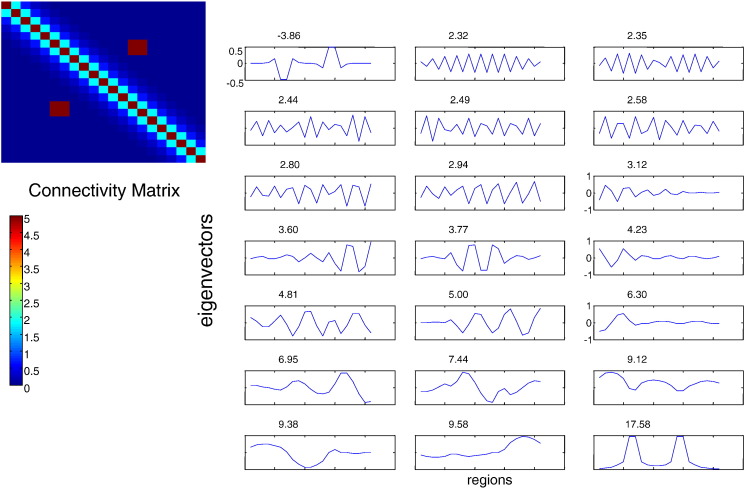
Symmetric connectivity matrix with two-point local connections and corresponding eigenvectors and eigenvalues (above each plot).

We now focus on the eigenmodes of connectivity configurations formulated in terms of inhomogeneous or translationally invariant matrices. We here confirm the analysis of earlier sections by performing numerical simulations: as predicted, translationally invariant matrices that underlie certain neural field models have a full spectrum of damped spatially periodic patterns (see Fig. 4). On the other hand, eigenmodes tend to be more localised as the degree of inhomogeneity increases. We destroyed the homogeneity by introducing two-point local connections, namely the off-diagonal connections in Fig. 5. This results in the appearance of bumps (localised activity) in the dominant mode depicted at the right bottom corner of this figure.

### Non-symmetric connectivity matrices

We here include a discussion of anatomical connectivity matrices of the sort found in the CoCoMac database (Stephan et al., 2001). This important database contains data from numerous tracing studies of the macaque brain and includes connectivity matrices that retain directionality features. These matrices are non-symmetric and comprise excitatory long range and excitatory and inhibitory short range connections. Cortical networks characterised by non-symmetric connectivity can exhibit an oscillatory and a non-oscillatory instability, the former depending critically on time-delays (Jirsa and Ding, 2004). We examined the impact of directed connections by repeating the numerical simulations described above but using an asymmetric anatomical connectivity.

**Fig. 6:**
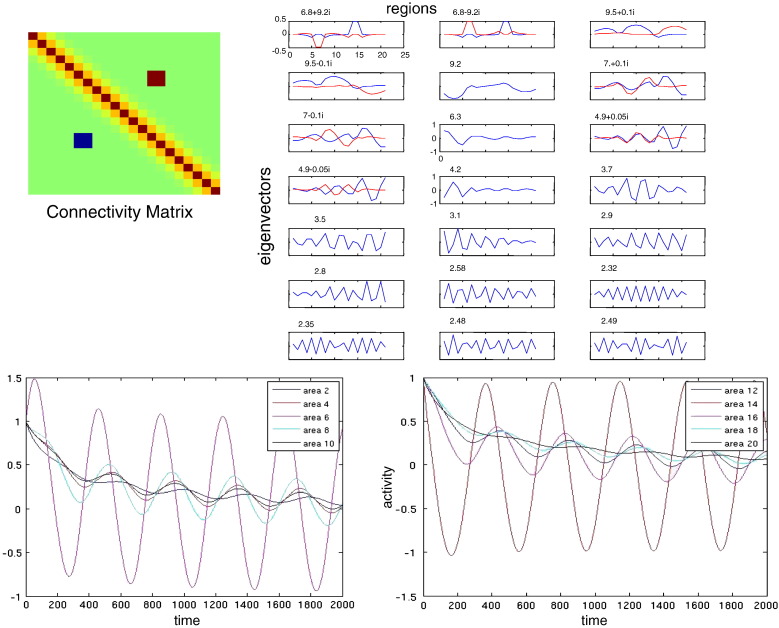
(Upper left:) Connectivity matrix involving homogeneous and short range two point connections (excitatory and inhibitory). (Right:) Corresponding eigenvectors where the real and imaginary parts are depicted in blue and red respectively. The relevant eigenvalue is depicted at the top of each panel. (lower left:) Areas 6 and 14 oscillate with a phase difference of *π*/2.

As in the symmetric case, resting state activity can show strong similarities with the principal eigenmode and, as expected, fluctuations around an oscillatory instability correspond to a complex-valued mode. In other words, the introduction of asymmetries into the connectivity matrix – in the form of local (reciprocal) excitatory and inhibitory connections depicted as red (*κ*_*ij*_ > 0) and blue (*κ*_*ij*_ < 0) two-point connections in Fig. 6 respectively – results in the appearance of complex eigenvalues and eigenmodes describing oscillatory cortical activity (see Fig. 6). Interestingly, the appearance of a complex principal eigenomode results in a spatial segregation of coherent activity; namely, the imaginary part of the eigenmodes corresponds to a second cortical clique that oscillates at the same frequency with the clique that corresponds to the real part, but with a phase difference of ± *π*/2. In other words, for the connectivity configuration of Fig. 6, resting state activity, dominated by the mode in the top left panel, involves regions that oscillate with a constant lag (see e.g. areas 6 and 14 in the bottom panel). This could provide a simple and elegant explanation for characteristic phase differences in coherent fluctuations observed in resting state fMRI (Biswal et al., 1995).

**Fig. 7:**
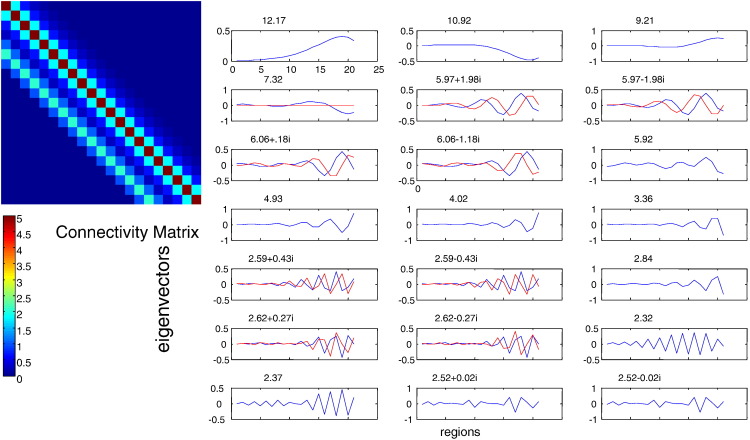
(Left:) Non-symmetric connectivity matrix with traits of translational invariance. (Right:) Corresponding eigenvectors.

On the other hand, the eigenmodes of translationally invariant but non-symmetric connectivity matrices do not show this behaviour and are, as expected, similar to the periodic modes seen under homogenous symmetric coupling (see Fig. 7). The corresponding eigenmodes are now complex (due to lack of symmetry) with real and imaginary parts resembling the eigenmodes of symmetric matrices biased towards part of the eigenspace as one may see by comparing Figs. 4 and 7.

### The DSI matrix

**Fig. 8:**
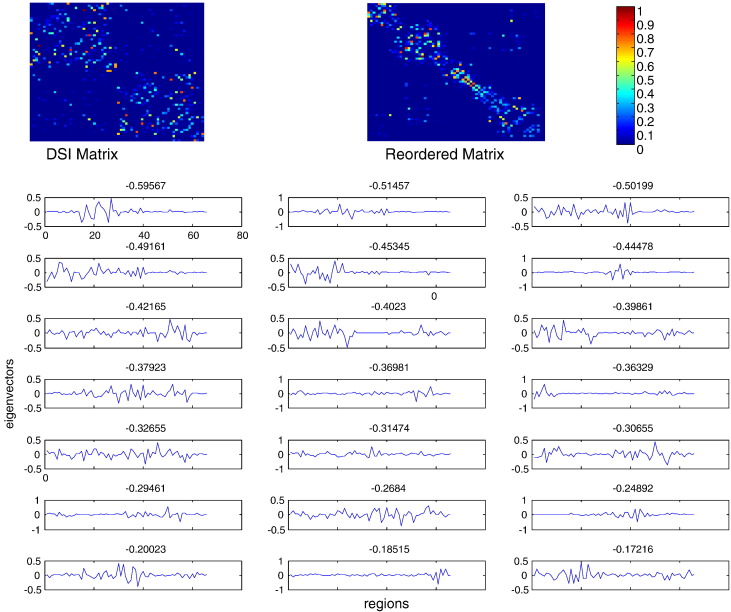
DSI matrix (top left), its reordered version to reveal optimal communities (top right) and corresponding eigenmodes and eigenvalues (bottom).

Our final simulations attempted to discover any evidence that would support the assumptions of homogenous lateral connectivity using the DSI anatomical connectivity matrix described in the first section. The idea here was that by expressing the principal eigenmodes over nodes that have been reordered to reveal their community structure, we might find evidence for fluctuations dominated by damped spatially periodic modes of the sort predicted under locally homogenous connectivity. As expected, a reordering of the DSI connectivity matrix motivated by the reordered versions of the exemplar matrices of Fig. 2, juxtaposes nodes belonging to the same communities and reveals band structures around the main diagonal.^7^ The corresponding eigenmodes exhibit a small amount of spatial periodicity, however they do not constitute evidence for a spectrum consisting of periodic patterns (see Fig. 8).

## Conclusion

This computational work has used integro-differential equations to model cortical dynamics under homogenous and inhomogeneous assumptions about anatomical connectivity. This form of model suggests that the eigenmodes of resting state activity (under local linear stability assumptions) should conform to the eigenmodes of the underlying anatomical connectivity. As expected, when this anatomical connectivity is homogeneous (or translationally invariant) the resulting modes consist of damped spatially periodic patterns. Numerical simulations confirmed these analytic results, showing that inhomogeneous connectivity destroyed such modes otherwise dominating resting state fluctuations. When using real (DSI) anatomical connectivity matrices, we found a similar absence of a spectrum of patterns of increasing spatial periodicity that would be predicted under homogeneity assumptions for fixed point mean field dynamics. This suggests that assumptions of translational invariance for such dynamics are probably not appropriate for modelling large-scale cortical activity, even when local communities or cliques can be identified with graph theory measures.

Clearly, the analyses in this paper may be too simple to completely dispense with the adoption of homogeneity assumptions for whole-brain or cortical dynamics. For example, we have only considered translational invariance in one dimension and excluded oscillatory mean field dynamics; however, equivalent invariances over the two dimensions of the cortical sheet may provide a more plausible model of endogenous fluctuations at resting state (Peter Robinson — personal communication) and can also give inhomogeneous correlations (Coombes et al., 2007; Robinson, 2006, 2012). Having said this, the results of our analysis speak to the adoption of general models of cortical networks that allow for arbitrary inhomogeneous coupling. Indeed, our current work on large stochastic dynamic causal models pursues this direction. One might then ask; what is the role of neural field theory in neuroimaging?

One compelling answer is that the local (mesoscopic) dynamics associated with (macroscopic) cortical sources (for either EEG or fMRI) may be usefully modelled with neural fields with constant coefficients in continuous media. In other words, generative or dynamic causal models of empirical data could comprise graphs where each node is, itself, such a neural field. The fundamental advantage of this sort of model is that the dynamics at each node can now be parameterised in terms of lateral connections (the neural field connectivity kernel). In this case, neural fields will provide local spatiotemporal dynamics where input to and output from each node will be considered independently of other nodes. Note that in this application of neural field theory, the spatial part of the spatiotemporal dynamics does not have to be observed directly – the neural field model simply provides a spatial model to explain observed temporal dynamics in the time or frequency domain. Future work using both invasive and non-invasive electrophysiology will illustrate the utility of neural field theory in this empirical setting.

## Footnotes

1 Other variants include inhomogeneous approaches to neural field theory (see e.g. Bressloff, 2003; Jirsa and Kelso, 2000).

2 It should be noted that sparseness does not necessarily imply inhomogeneity and these two terms capture different aspects of connectivity. Also, the work of anatomists such as Braitenberg and Schüz (1998) has revealed that the connection kernel in the cortex exhibits strong homogeneity traits. Also, Henderson and Robinson (2011) has shown that highly nonuniform-looking connection matrices, even with high modularity measures, can result from completely homogeneous connectivity in 2D.

3 Localised modes can appear as a result of superposed damped spatially periodic modes. This is possible in theory (see e.g. Dunford and Schwartz, 1988), but in real world situations it is difficult to realise, as a fully localised solution would require the interference of many correctly phased modes.

4 It can be shown that this class includes functions in a space spanned by exponential polynomials, namely functions of the form $x^{j-1}e^{-r_{i}x}$ (for examples see Abramowitz and Stegun, 1972).

5 For limitations of such equations (see Bojak and Liley, 2010).

6 But see footnote 3.

7 It should be noted that a band structure around the main diagonal is exactly what is expected when a totally homogeneous 2D cortex is labeled with 1D raster-ordered labels (see also Robinson, 2012).
